# Effects of a combined nature-based and audio-based virtual mindfulness intervention on stress and wellbeing of COVID-19 healthcare workers: a randomized controlled trial

**DOI:** 10.7717/peerj.19109

**Published:** 2025-05-23

**Authors:** Polycarpe Bagereka, Rezvan Ameli, Ninet Sinaii, Marcelli Cristine Vocci, Angelina Mangiardi Coulter, Michael Neustedter, Ann Berger

**Affiliations:** 1 Pain and Palliative Care Service, National Institutes of Health, Bethesda, MD, United States of America; 2 Biostatistics and Clinical Epidemiology Service, National Institutes of Health, Bethesda, MD, United States of America; 3 First Descents, Denver, CO, United States of America

**Keywords:** COVID-19, Healthcare workers, Wellbeing, Mindfulness, Nature-based therapy, Audio-based intervention, Palliative care

## Abstract

**Background:**

The global COVID-19 pandemic and its subsequent transition to an endemic phase has highly increased psychosocial distress among healthcare workers. This chronic stress may culminate into burnout, which has been associated with impaired patient care and increased medical errors. Exposure to nature-based programs have been shown to reduce levels of perceived stress and promote well-being. However, benefits derived from nature programs can be short-lived. Mindfulness-based interventions represent a promising additive option to enhance benefit. The present study proposes to combine a nature-based program with an audio-based mindfulness intervention to address stress and promote psychosocial-spiritual wellbeing in COVID-19 healthcare workers.

**Methods:**

Between June 2021 and October 2023, 78 healthcare workers were randomized into a Nature only group (Nature), a Combined Nature-Mindfulness group (Combined), and a control group (Control), with 19, 16, and 18 subjects completing the study, respectively (23–46 years, mean = 35, SD = 6). The nature program was a three or five-day retreat during which participants engaged in rock climbing, surfing or kayaking. The virtual audio mindfulness intervention was a 10-day program offered online and included mindful breathing, body scan, and loving-kindness meditation. Participants completed self-administered assessments at three or four time points during the study, including at baseline and following study procedures. Assessments included a measure of stress, the Perceived Stress Scale, and a measure of wellbeing, the National Institute of Health Healing Experience of All Life Stressors (NIH-HEALS).

**Results:**

The mean baseline Perceived Stress Scale score was 21.2 ± 3.2 in the Combined group, 22.3 ± 2.8 in the Nature group, and 23.2 ± 3.3 in the Control group. Perceived Stress Scale levels did not change following nature-based and mindfulness-based interventions. The mean baseline NIH-HEALS score was 123.1 ± 19.4 in the Combined group, 118.1 ± 17.1 in the Nature group, and 114.3 ± 17.8 in the Control groups. NIH-HEALS scores increased in both the Nature and Combined groups when compared to the Control group. At follow-up, the Combined group maintained improvements in wellbeing when compared to the Nature group.

**Conclusions:**

Findings suggest that nature-based programs can be used to improve overall wellbeing among COVID-19 healthcare workers. Additionally, integrating audio-based mindfulness practices to these programs may enhance their benefits. Notably however, these interventions may not effectively reduce perceived stress among COVID-19 healthcare workers. Due to diminished power of the present study, further research is needed to validate and refine the present findings.

## Introduction

The COVID-19 pandemic has contributed to psychosocial distress experienced by healthcare workers. The context surrounding the care of patients with COVID-19 may involve witnessing patient death, death of colleagues, and the distress of exposing oneself and family to the virus (Huang et al., 2020; Murthy, 2022; Santarone, McKenney & Elkbuli, 2020). Healthcare workers (HCWs) working under these conditions can experience increased levels of stress, trauma, and psychosocial-spiritual distress (Andhavarapu et al., 2022; Cenat et al., 2021; Lee et al., 2023; Lu et al., 2020; Muehlhausen, 2021; Shanafelt, Ripp & Trockel, 2020; Wu et al., 2021). Such chronic stress and spiritual distress may culminate into burnout, which has been associated with impaired patient care and increased medical errors, posing risks to patient health (Fahrenkopf et al., 2008; Holland & Neimeyer, 2005; Jungmar Ridell & Orvelius, 2023; Muehlhausen, 2021; West et al., 2006).

Psychosocial-spiritual wellbeing is related to overall wellbeing and associated with improved coping ability and quality of life. Recent studies document the benefits of mindfulness and nature-based interventions for mental health and wellbeing, including their potential to mitigate the adverse effects of stress, depression, and anxiety (Coventry et al., 2021; Khoury et al., 2015; Labib, Lindley & Huck, 2020; Poulsen, Stigsdotter & Davidsen, 2018). Nature-based programs typically use outdoor activities such as rock climbing, surfing, and kayaking to promote wellbeing and personal growth (Herring, Knowles & Alschuler, 2021). These programs demonstrate efficacy in decreasing symptoms of depression, alienation, and improving self-efficacy (Rosenberg et al., 2014; Zebrack, Kwak & Sundstrom, 2017). Notably, however, benefits derived from nature-based programs can be short-lived and may require additional interventions to strengthen and prolong their efficacy (Kotera, Richardson & Sheffield, 2022).

Mindfulness interventions are evidence-based (Green & Kinchen, 2021; Melnyk et al., 2020; Yang et al., 2023) and represent a promising option in combination with nature programs to enhance benefit (Menardo et al., 2022). Mindfulness is defined as the awareness that emerges through paying attention, on purpose, in the present moment, and non-judgmentally to the unfolding of experience (Kabat-Zinn, 1990). Mindfulness-based interventions are highly correlated with psychosocial-spiritual wellbeing (Bagereka et al., 2023) and are reported to alleviate symptoms associated with stress and trauma (Lomas et al., 2019). Among HCWs, mindfulness-based interventions, including brief therapies, have been shown to reduce work-related stress, anxiety, and burnout while improving measures of wellbeing including job satisfaction, empathy, and fulfillment (Ameli et al., 2020; Chmielewski, Łoś & Łuczyński, 2021; Ghawadra et al., 2020; Goldhagen et al., 2015; Green & Kinchen, 2021; Krasner et al., 2009).

The present study proposes to combine a nature-based program with an audio-based mindfulness intervention to address stress and psychosocial-spiritual wellbeing. To investigate the efficacy of this combined intervention, we designed a study with three groups: the combined Nature-Mindfulness group (Combined), the Nature-only group (Nature), and a control group (Control). This design allows us to evaluate the specific and combined effects of nature-based activities and mindfulness practices on the wellbeing of healthcare workers. We hypothesize that HCWs in the Combined group will have greater reduction in stress and improvement in wellbeing when compared to the Nature and the Control groups. Similarly, we expect HCWs in either Nature group to have greater reduction in stress and improvement in wellbeing when compared to the Control group.

## Materials & Methods

This randomized controlled study was approved by the National Institutes of Health (NIH) Institutional Review Board (IRB) at the Office of Human Subject Research Protection (OHSRP) approval number 000192 (NCT04846790). Verbal and written consent were obtained from each participant prior to enrollment. Data were collected anonymously and were deidentified, and study procedures were noninvasive. Participation was voluntary and all were informed that they could withdraw from the study at any time.

### Participants and recruitment

Active COVID-19 HCWs were recruited in collaboration with First Descents (FD). FD, founded in 2001, is a Colorado based non-profit 501(c)(3) organization which provides free outdoor adventure activities to young adults impacted by cancer and other serious health conditions. FD offered their nature-based programs to HCWs including physicians, nurses, and others during the COVID-19 pandemic from June 2021 to October 2023.

Healthcare worker applicants to FD nature programs were informed about the study. Interested individuals were screened for eligibility by phone by an NIH research team member. If qualified, they were given a unique number to sign into the database program, REDCap (Research Electronic Data Capture)—a secure, web-based software used for creating and managing online surveys and databases, where study data were captured. Participants also received additional study description and materials including a written consent document to complete.

To be eligible for the study, participants had to be 18 years or older and an HCW who had provided care to patients with or suspected of COVID-19. They were required to have internet access and a compatible device to complete the online questionnaires. Additionally, participants needed to be fluent in English and capable of providing informed consent independently. Exclusion criteria included presence of an acute psychiatric condition, non-modifiable hearing impairments, and/or lack of devices or internet access to complete or listen to study-related online audio recordings.

### Study design and procedures

The study included three groups: a Combined Nature-Mindfulness group (Combined), a Nature-only group (Nature), and a control group (Control). The Combined group participated in both the nature program and the mindfulness program. The Nature group participated only in the nature program. The Control group did not participate in either program while completing the study assessments but later participated in the FD nature program.

Eligible participants were randomized into one of the three, Combined, Nature, or control groups. The randomization scheme was based on block sizes of six, with 2:2:2 random assignments into each group. Participants completed a demographic questionnaire. They also completed self-administered assessments at three or four time points, depending on group and per study design. REDCap automated prompts for tasks and assessments were emailed throughout the study. Each assessment took about 40 min to complete.

**Figure 1:**
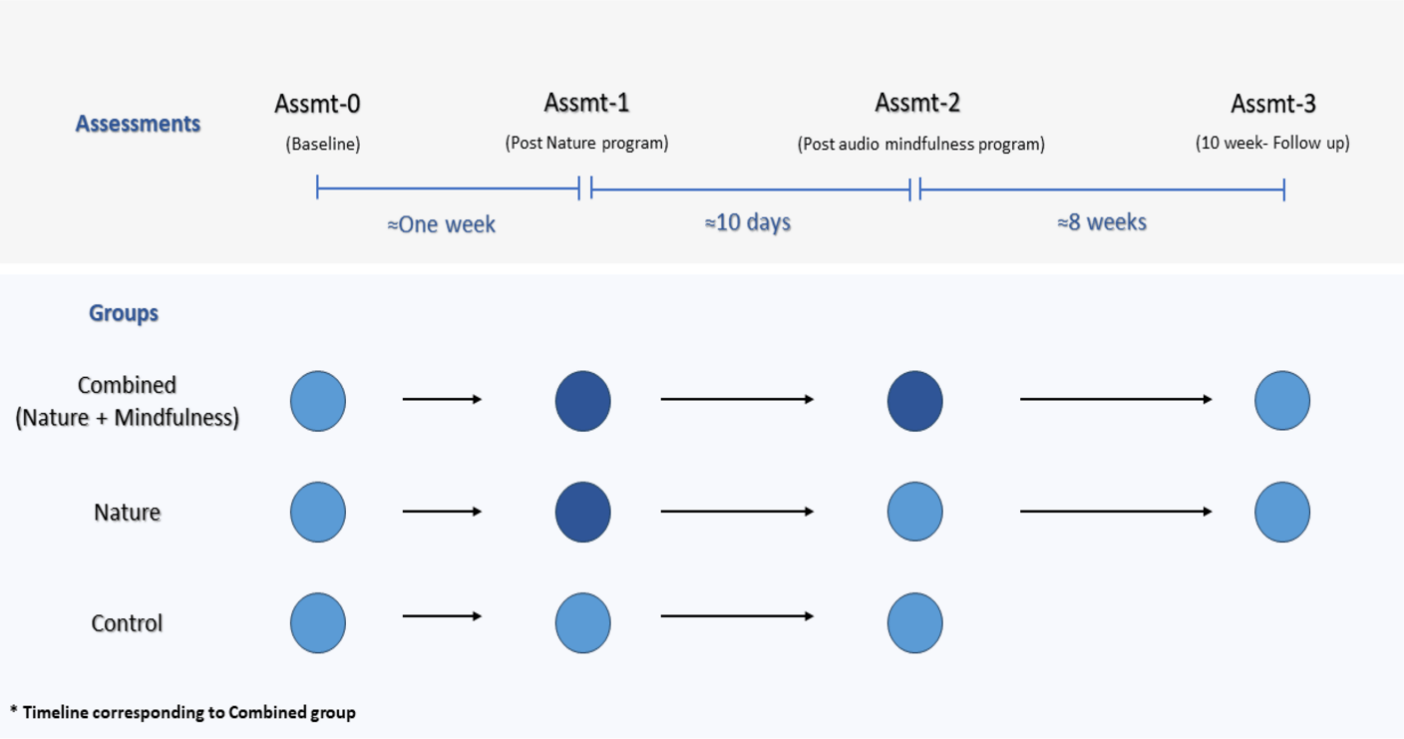
Study design. Circles represent applicable assessments; dark circles reflect interventions (nature-based program, and nature plus audio-based mindfulness program).

The Combined group completed a total of four assessments. Their baseline assessment (Assmt-0) was at least one week before starting the nature program. The day after the nature program had ended, participants completed another set of assessments, Assmt-1. They then began a 10-day audio mindfulness program, followed immediately by Assmt-2. The Combined group also completed a two-month follow-up assessment, Assmt-3. Throughout the eight weeks, participants received weekly reminders to engage in mindfulness activities that they had learned during the 10-day mindfulness program (see Fig. 1, study design).

The Nature group also completed a total of four assessments. Assmt-0 was at least one week before starting the nature program. The day after the nature program had ended, participants completed Assmt-1. Ten days later, they completed Assmt-2. A two-month follow-up assessment (Assmt-3) was also administered. Throughout the eight weeks, participants received short weekly surveys to monitor engagement (see Fig. 1).

Participants in the Control group completed three assessments: Assmt-0, Assmt-1, and Assmt-2. Assmt-1 was completed one week after Assmt-0, and Assmt-2 was completed ten days after Assmt-1 (Fig. 1). All assessments were administered before participation in the nature program. Assmt-2 (see Fig. 1) time point was the study primary endpoint for all participants. Follow up data were collected for the intervention (Combined or Nature) groups only. The 10-week follow-up assessment period was deemed an inappropriate wait time for Control group participants who were eager to participate in the FD nature. They were offered the mindfulness program on their own if they chose to use it following their study participation.

### Nature program

The nature program was offered by FD, which has long provided similar programs to young adults with cancer and other serious health conditions with positive results (Rosenberg et al., 2014; Zebrack, Kwak & Sundstrom, 2017). The one-time program was a three or five-day retreat in a nature-rich environment during which participants engaged in rock climbing, surfing or kayaking. The program was in-person and was offered on different dates and at several locations throughout the USA. Participants enrolled in a program and location of their preference. There was no cost to attend, and meals and lodging were included. Special precautions against COVID-19 transmission were also implemented.

Each program consisted of 12–14 participants. Participants were sometimes broken into groups during the activity to provide more instruction, time, and attention to each participant. Participants shared these experiences together and interacted with each other throughout the program.

### Program facilitators

Each program included facilitators who guided participants through their activity (rock climbing, surfing, or kayaking). Facilitators provided participants with all the necessary gear and equipment such as helmets, personal flotation devices, harnesses, climbing shoes, wetsuits, kayaks, paddles, and proper instruction for their use.

In addition to the facilitators, each program was supported with a lead staff, chefs, and volunteers. Lead staff were employees of FD and were trained to facilitate the “First Descents Experience” such as the overall flow of activities and group discussions. Volunteers, who were previous FD participants, served as general program support, photographer, or medical support.

The lead staff and outfitters for each program were responsible for assessing weather conditions and group needs each day to determine the best location such as climbing or river location and safe activity recommendations.

### COVID-19 precautions

COVID-19 precautions were implemented at each program and were based on ongoing recommendations of the Centers for Disease Control and Prevention (CDC).

### Audio mindfulness program

In addition to the nature intervention, the Combined group completed an audio mindfulness program. This intervention was 10 days long and was offered online. The program was developed by an experienced NIH mindfulness teacher and investigator (RA). It included a manual that described mindfulness and the elements of the 10-day practice. The manual was available to the participants for download. Each day the participant received a notification that a new mindfulness audio was ready for listening. These recordings varied in length from 10 to 25 min. Mindfulness exercises included mindful breathing, body scan, and loving-kindness meditation. In addition, instructions for mindful walking, mindful eating, and mindful hand-hygiene were provided. Participants could listen to the daily audio as many times as they wished but could not listen to the next day’s content.

• **Day one**: Introduction to the concept of mindfulness followed by a short practice.
• **Day two**: A focus on the indispensable elements of mindfulness, including intention, attention, attitude, and awareness. Participants engaged in a mindful breathing practice incorporating sounds.
• **Day three**: Attitudinal foundations of mindfulness, including nonjudgment, patience, beginner’s mind, trust, non-striving, acceptance and letting go followed by a mindful breathing practice.
• **Day four**: Participants practiced the body scan on the floor, lying down on a mat or blanket. Mindfulness principles were incorporated in this practice.
• **Day five**: Practice of mindful breathing and the body scan in a seated position.
• **Day six**: Handling challenges to concentration such as fatigue, craving, and irritation. Participants learned to recognize a wandering mind, refocus attention to the anchor, and accept rising emotions and changing mental states.
• **Day seven**: Mindfulness meditation with various anchors.
• **Day eight**: Applications of mindfulness through mindful walking, mindful eating, and mindful consumption in general.
• **Day nine**: A focus on application of mindfulness to COVID-19, including mindful hand hygiene.
• **Day ten**: Loving-kindness and compassion as anchors for meditation.

### Outcome measures

The Perceived Stress Scale (PSS) was used to measure stress level and was the primary outcome measure. PSS is a 10-item self-report scale and is scored on a 0–4 scale ranging from never to very often. This is a widely used and validated measure with Cronbach’s alpha ranging between 0.75 and 0.91 (Cohen, Kamarck & Mermelstein, 1983; Nielsen et al., 2016).

The National Institutes of Health–Healing Experience of All Life Stressors (NIH-HEALS), a secondary outcome measure, is a 35-item self-report questionnaire on a 5-point Likert scale developed by the NIH Clinical Center Pain and Palliative Care Service. NIH-HEALS is a measure of healing that assesses an individual’s psycho-social-spiritual mechanisms for coping during life’s difficult situations and/or life-limiting challenges. The NIH-HEALS is valid and reliable (Cronbach’s alpha = 0.89, split-half reliability=0.95) (Ameli et al., 2018; Sloan et al., 2017).

### Sample size calculation and statistical analysis

A detection of a 4.0 difference in the PSS score, the primary outcome on which sample size was based, was determined to be a meaningful change. Sample size estimation was based on a two-sided *t*-test, with unequal variances, power at 0.85, delta of 4.0, and standard deviations of 4.0. Alpha was set at 0.0167 to account for the comparison of three groups. This yielded a sample size of 26 per group. Considering an expected 15% dropout or lost-to-follow-up rate, a total of 30 participants per group, resulting in 90 participants in total, would produce sufficient power. However, due to challenges in recruitment and the short program duration from June 2021–Oct 2023, the required sample size was not fulfilled. Thus, the power to detect the initial difference dropped to 58%. Despite the low statistical power, significant changes were observed in NIH-HEALS. Therefore, definitive conclusions cannot be drawn regarding the PSS as it did not show a significant difference.

Generalized linear mixed models for repeated measures were used for analysis, considering the baseline levels and within-group comparisons across time. Data were analyzed and are presented as comparisons among the three groups as well as by intervention (Combined and Nature) *versus* Controls. The models were tested for covariance matrix fit. Data were also assessed for the potential confounding effects of other covariates, such as participant age and sex, profession, and level and type of exposure to COVID-19. The final models included group, time, and the interaction for group and time, using unstructured covariance matrix. *Post-hoc* analyses were carried out for specific pairwise comparisons (*e.g.*, Combined *vs* Nature, Nature *vs* Control), adjusting for multiple comparisons using the Bonferroni method.

## Results

**Figure 2:**
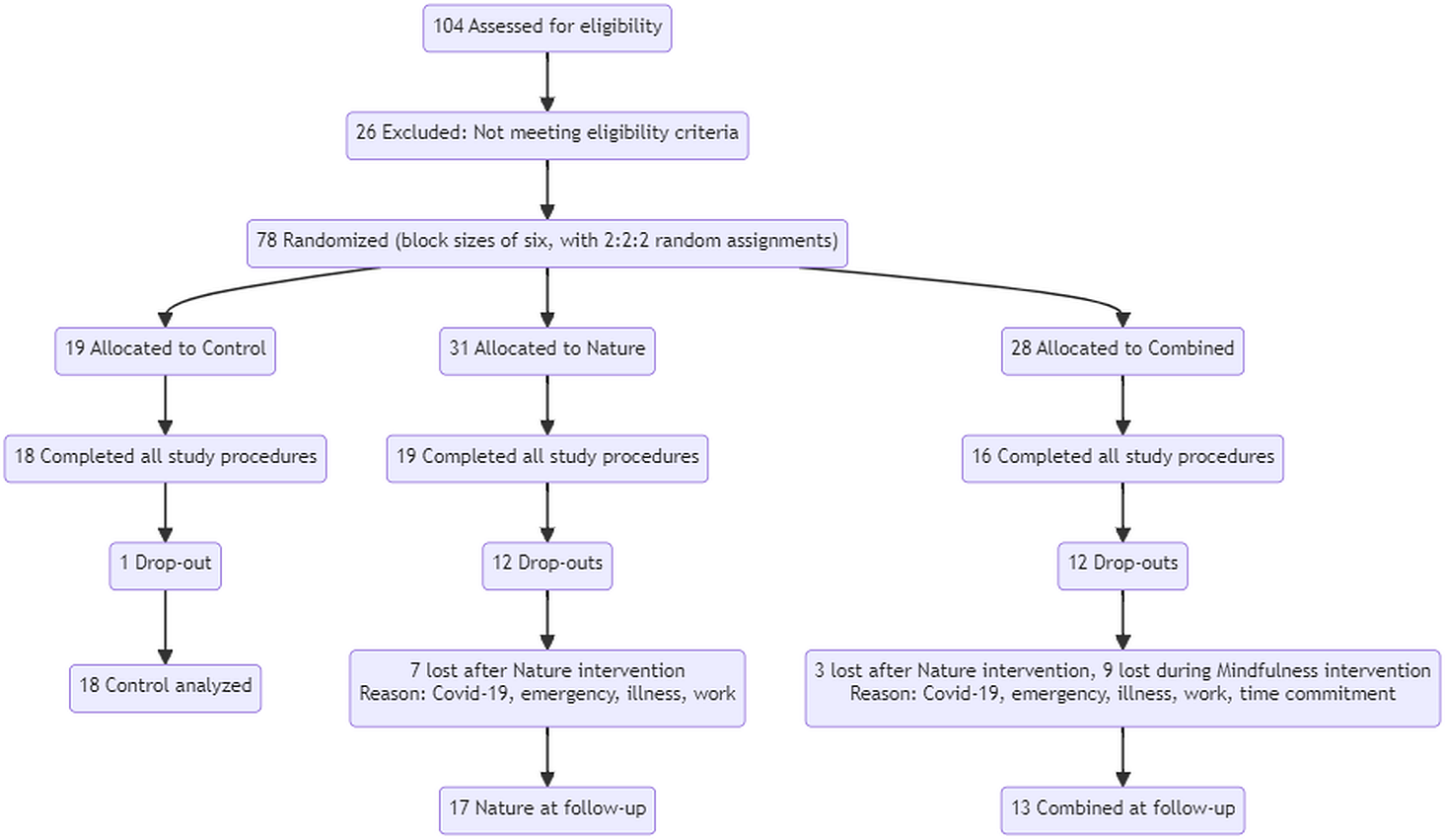
CONSORT flow diagram.

Seventy-eight subjects were randomized to participate in the study. Fifty-three participants completed the study, including 16 in the Combined group, 19 in the Nature group, and 18 in the Control group. There was a comparable dropout rate for the two intervention groups with similar reasons for dropping out (see Fig. 2, the flow diagram).

The mean age was 35.7 ± 6.2 years. Participants indicated their gender as male (11%) or female (88%). Other gender identifications were not made. Participants were White (68%), Asian (21%), Black (2%), or Other (9%). The vast majority (70%) of participants worked in a hospital setting, and indicated their occupation as Nurse (55%), Physician (13%), Nurse Practitioner (7%), Physician Assistant (6%), or Social Worker (6%). All had exposure to COVID-19, either directly (77%), indirectly (4%), or both (19%). Participants reported their pandemic stress levels as no stress (0%), mild (0%), moderate (45%), severe (34%), very severe (13%), or extreme (7%). Further description of participant demographics is detailed in Table 1.

**Table 1:** Demographic and baseline characteristics of a cohort of COVID-19 healthcare workers.

Characteristic	Total (*n* = 53)	Combined (*n* = 16)	Nature (*n* = 19)	Control (*n* = 18)
Age, in years, mean ±SD	35.7 ± 6.2	38.7 ± 5.2	34.8 ± 7.1	33.9 ± 5.2
Gender				
Male	6 (11.3%)	2 (12.5%)	2 (10.5%)	2 (11.1%)
Female	47 (88.7%)	14 (87.5%)	17 (89.5%)	16 (88.9%)
Race				
White	36 (67.9%)	9 (56.3%)	15 (79.0%)	12 (66.7%)
Asian	11 (20.8%)	4 (25.0%)	3 (15.8%)	4 (22.2%)
Mixed/Other/Unknown	5 (9.4%)	2 (12.5%)	1 (5.3%)	2 (11.1%)
Black or African American	1 (1.9%)	1 (6.3%)	0	0
American Indian/Alaska Native	0	0	0	0
Native Hawaiian/Pacific Islander	0	0	0	0
Ethnicity				
Hispanic/Latinx	6 (11.3%)	1 (6.3%)	4 (21.1%)	1 (5.6%)
Non-Hispanic/Latinx	46 (86.8%)	15 (93.8%)	15 (79.0%)	16 (88.9%)
Marital status				
Single	21 (39.6%)	8 (50.0%)	8 (42.1%)	5 (27.8%)
Married	19 (35.9%)	6 (37.5%)	6 (31.6%)	7 (38.9%)
Divorced/Separated	6 (11.3%)	2 (12.5%)	3 (15.8%)	1 (5.6%)
Widowed	1 (1.9%)	0	0	1 (5.6%)
Living with partner	6 (11.3%)	0	2 (10.5%)	4 (22.2%)
Religion				
Agnostic	14 (26.4%)	5 (31.3%)	4 (21.1%)	5 (27.8%)
Atheist	5 (9.4%)	0	2 (10.5%)	3 (16.7%)
Buddhism	0	0	0	0
Christianity	19 (35.9%)	6 (37.5%)	7 (36.8%)	6 (33.3%)
Hinduism	1 (1.9%)	1 (6.3%)	0	0
Islam	2 (3.8%)	0	0	2 (11.1%)
Judaism	2 (3.8%)	1 (6.3%)	1 (5.3%)	0
Other/Prefer not to answer	10 (18.9%)	3 (18.8%)	5 (26.3%)	2 (11.1%)
Occupation				
Certified Nursing Assistant	1 (1.9%)	0	0	1 (5.6%)
Dietician	1 (1.9%)	1 (6.3%)	0	0
Intern/Resident/Fellow	1 (1.9%)	0	0	1 (5.6%)
Medical Assistant	1 (1.9%)	0	0	1 (5.6%)
Medical Student	0	0	0	0
Nurse	29 (54.7%)	7 (43.8%)	13 (68.4%)	9 (50.0%)
Nurse Practitioner	4 (7.6%)	0	2 (10.5%)	2 (11.1%)
Paramedic/Emergency Medical	0	0	0	0
Technician				
Physician	7 (13.2%)	3 (18.8%)	0	4 (22.2%)
Physician Assistant	3 (5.7%)	2 (12.5%)	1 (5.3%)	0
Respiratory Therapist	0	0	0	0
Social Worker	3 (5.7%)	2 (12.5%)	1 (5.3%)	0
Other	3 (5.7%)	1 (6.3%)	2 (10.5%)	0
Service setting, all applicable				
Clinic	13 (24.5%)	7 (43.8%)	2 (10.5%)	4 (22.2%)
Hospice	1 (1.9%)	0	1 (5.3%)	0
Hospital	37 (69.8%)	11 (68.8%)	13 (68.4%)	13 (72.2%)
Hospital Emergency Room	9 (17.0%)	1 (6.3%)	4 (21.1%)	4 (22.2%)
Nursing Home	3 (5.7%)	1 (6.3%)	1 (5.3%)	1 (5.6%)
Patient Residence	4 (7.6%)	2 (12.5%)	2 (10.5%)	0
Urgent Care	3 (5.7%)	1 (6.3%)	0	2 (11.1%)
Other	8 (15.1%)	5 (31.3%)	1 (5.3%)	2 (11.1%)
COVID-19 exposure				
Direct	41 (77.4%)	10 (62.5%)	17 (89.5%)	14 (77.8%)
Indirect	2 (3.8%)	0	1 (5.3%)	1 (5.6%)
Both	10 (18.9%)	6 (37.5%)	1 (5.3%)	3 (16.7%)
Frequency of COVID-19 exposure				
Rarely (every few months)	8 (15.1%)	3 (18.8%)	3 (15.8%)	2 (11.1%)
Occasionally (every few weeks)	16 (30.2%)	6 (37.5%)	5 (26.3%)	5 (27.8%)
Frequently (weekly)	8 (15.1%)	5 (31.3%)	1 (5.3%)	2 (11.1%)
Often (few days a week)	14 (26.4%)	2 (12.5%)	7 (36.8%)	5 (27.8%)
Daily	7 (13.2%)	0	3 (15.8%)	4 (22.2%)
Infected with COVID-19	29 (54.7%)	6 (37.5%)	8 (42.1%)	15 (16.7%)
Vaccinated for COVID-19	51 (96.2%)	15 (93.8%)	19 (100.0%)	17 (94.4%)
Most stressful aspect of working with patients with COVID-19				
Concern about getting infected	3 (5.7%)	1 (6.3%)	0	2 (11.1%)
Concern about infecting others	11 (20.8%)	6 (37.5%)	2 (10.5%)	3 (16.7%)
Social distancing/isolation	3 (5.7%)	1 (6.3%)	0	2 (11.1%)
COVID-19 illness in someone close	4 (7.6%)	2 (12.5%)	1 (5.3%)	1 (5.6%)
Death due to COVID-19 in someone close	3 (5.7%)	2 (12.5%)	1 (5.3%)	0
Witnessed or heard about patient death from COVID-19	4 (7.6%)	1 (6.3%)	0	3 (16.7%)
Insufficient support from workplace	1 (1.9%)	1 (6.3%)	0	0
Pressures of keeping up/following new procedural guidelines or use of new technology in caring for patients with COVID-19	2 (3.8%)	0	1 (5.3%)	1 (5.6%)
Constant news and statistics regarding COVID-19	0	0	0	0
Emotional strain, feeling overwhelmed, physical exhaustion, COVID fatigue	21 (39.6%)	2 (12.5%)	14 (73.7%)	5 (27.8%)
Vaccination status (in self, in others, variants, availability)	0	0	0	0
Other	1 (1.9%)	0	0	1 (1.9%)
Level of stress from COVID-19 pandemic				
No stress	0	0	0	0
Mild	0	0	0	0
Moderate	24 (45.3%)	9 (56.3%)	9 (47.4%)	6 (33.3%)
Severe	18 (34.0%)	6 (37.5%)	7 (36.8%)	5 (27.8%)
Very severe	7 (13.2%)	1 (6.3%)	1 (5.3%)	5 (27.8%)
Extreme	4 (7.6%)	0	2 (10.5%)	2 (11.1%)
Since COVID-19 pandemic, the meaning and internal satisfaction with work has:				
Decreased	35 (66.0%)	10 (62.5%)	13 (68.4%)	12 (66.7%)
Stayed the same	10 (18.9%)	4 (25.0%)	2 (10.5%)	4 (22.2%)
Increased	8 (15.1%)	2 (12.5%)	4 (21.1%)	2 (11.1%)
Current level of social support				
No support	0	0	0	0
Some support	16 (30.2%)	6 (37.5%)	4 (21.1%)	6 (33.3%)
Good support	25 (47.2%)	4 (25.0%)	11 (57.9%)	10 (55.6%)
Excellent support	12 (22.6%)	6 (37.5%)	4 (21.1%)	2 (11.1%)
Previous experience with mindfulness and meditation				
No experience	8 (15.1%)	1 (6.3%)	5 (26.3%)	2 (11.1%)
Some experience	39 (73.6%)	10 (62.5%)	13 (68.4%)	16 (88.9%)
A lot of experience	6 (11.3%)	5 (31.3%)	1 (5.3%)	0
In those with experience, frequency of mindfulness practice (*n* = 44)				
Occasionally	33 (75.0%)	9 (60.0%)	10 (71.4%)	14 (93.3%)
Frequently	7 (15.9%)	4 (26.7%)	2 (14.3%)	1 (6.7%)
Daily	4 (9.1%)	2 (13.3%)	2 (14.3%)	0

**Notes.**

Data are *n*(%); counts and percentages may not add to 100% due to rounding or missing/unknown responses. Randomized groups were combined Nature-Mindfulness (Combined), Nature-only (Nature), and Control groups.

**Figure 3:**
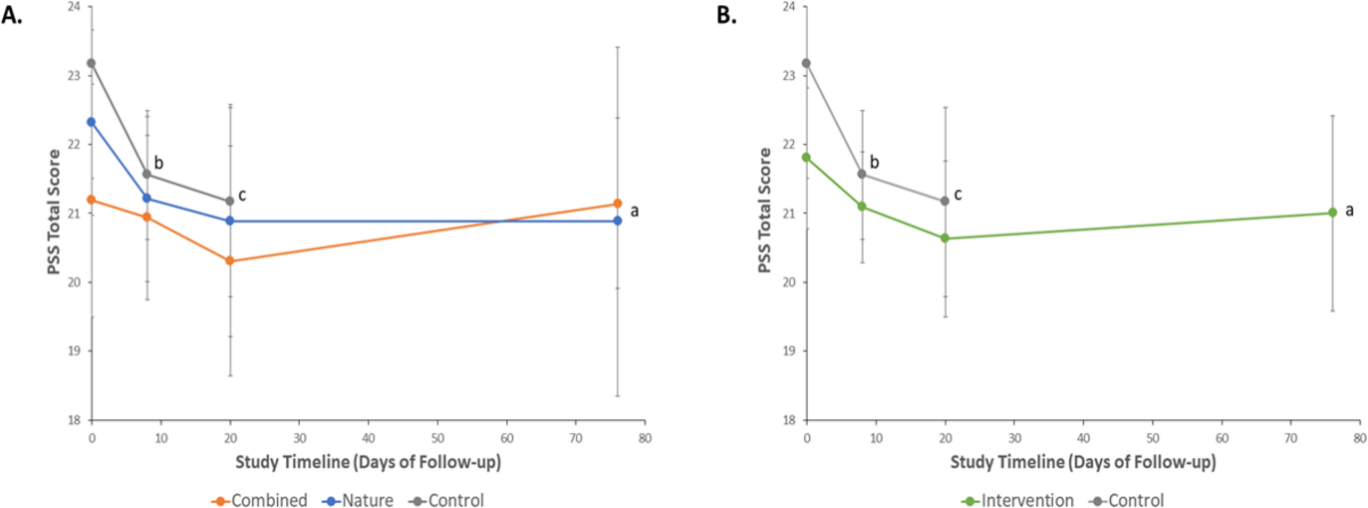
Changes in Perceived Stress Scale (PSS) total scores over time following nature-based and audio-based mindfulness interventions, or control. Results are from repeated measures analyses using generalized linear mixed models with between- and within-group comparisons. Data are mean scores ± 95% Confidence Intervals. Multiplicity adjusted *p*-values from pairwise comparisons utilized the Bonferroni method. (A) PSS scores over time by randomization group. ^a^*P* = 0.36 for overall group differences across time; *P* = 0.045 for overall time effect. ^b^In the control group, baseline (Assmt-0) *vs* each of Assmt-1 (corresponding to after nature-based program; adj *P* = 0.052), and ^c^Assmt-2(corresponding to after nature-based and audio-based mindfulness programs (Assmt-2; adj *P* = 0.057). (B) PSS scores over time in the Intervention (aggregated Combined and Nature) and Control groups. ^a^*P* = 0.20 for overall group differences across time; *P* = 0.026 for overall time effect. ^b^In the control group, baseline (Assmt-0) *vs* each of Assmt-1 (corresponding to after nature-based program; adj *P* = 0.051), and ^c^Assmt-2 (corresponding to after nature-based and audio-based mindfulness programs; adj *P* = 0.054).

The mean baseline PSS score, the primary outcome measure, was 21.2 ± 3.2 in the Combined, 22.3 ± 2.8 in the Nature, and 23.2 ± 3.3 in the Control groups (Table 2). Following Assmt-2, the primary endpoint, PSS score was 20.3 ± 3.1 in the Combined, 20.9 ± 3.5 in the Nature, and 21.2 ± 2.8 in the Control groups. PSS at follow-up, the secondary endpoint, was 21.1  ± 2.1 in the Combined and 20.9 ± 4.9 in the Nature groups (Table 2). Notably, overall global tests of significance showed a *p*-value of 0.36 among the group and 0.045 across time, indicating no substantial change among the groups but suggesting some change over time (Fig. 3A).

**Table 2:** Primary and secondary endpoints at study intervals in a cohort of COVID-19 healthcare workers. Randomized groups were Nature-Mindfulness (Combined), Nature-only (Nature), and Control groups. Sample sizes are listed where data were missing. Assessment 3 was not applicable to the Control group.

	Combined (*n* = 16)	Nature (*n* = 19)	Control (*n* = 18)	Global *P*-Value^a^
PSS				
Total score, mean ± SD				
Assmt-0	21.2 ± 3.2	22.3 ± 2.8	23.2 ± 3.3	0.36 (group effect)
Assmt-1	20.9 ± 2.2	21.2 ± 2.5	21.6 ± 1.9	0.045 (time effect)
Assmt-2^*^	20.3 ± 3.1	20.9 ± 3.5	21.2 ± 2.8	0.84 (group x time)
Assmt-3	21.1 ± 2.1	20.9 ± 4.9	(n/a)	
	(*n* = 14)	(*n* = 17)		
NIH-HEALS				
Total score, mean ± SD				
Assmt-0	123.1 ± 19.4	118.1 ± 17.1	114.3 ± 17.8	0.044 (group effect)
Assmt-1	128.9 ± 18.8	123.2 ± 19.0	111.4 ± 17.1	0.067 (time effect)
Assmt-2^*^	129.7 ± 19.7	126.8 ± 21.3	112.0 ± 15.3	0.028 (group x time)
Assmt-3	131.3 ± 21.2	120.4 ± 23.9	(n/a)	
	(*n* = 13)			

**Notes.**

Abbreviations NIH-HEALS National Institutes of Health –Healing Experience of All Life Stressors (NIH-HEALS) (Ameli et al., 2018; Sloan et al., 2017); PSS, Perceived Stress Scale (Cohen, Kamarck & Mermelstein, 1983; Nielsen et al., 2016)

a *P*-values are from the overall global tests of significance of each of the fixed effects (Type 3 test) from generalized linear mixed models for repeated measures.

* Primary outcome.

**Figure 4:**
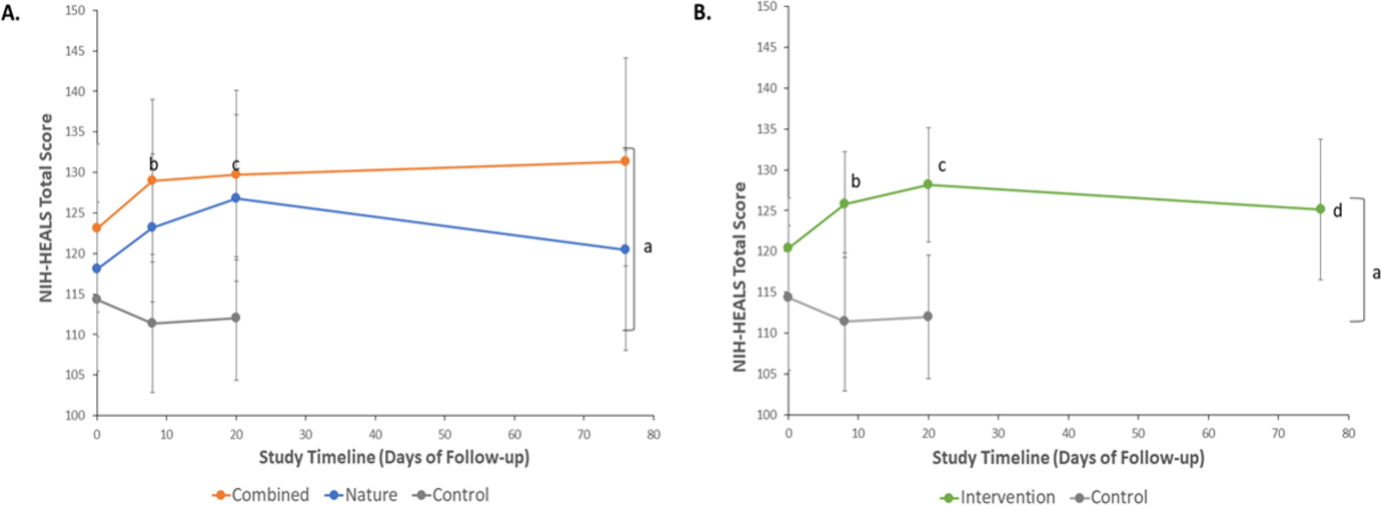
Changes in NIH-HEALS total scores over time following nature-based and audio-based mindfulness interventions, or Control. Results are from repeated measures analyses using generalized linear mixed models with between- and within-group comparisons. Data are mean scores ± 95% Confidence Intervals. Multiplicity adjusted *p*-values from pairwise comparisons utilized the Bonferroni method. (A) NIH-HEALS scores over time by randomization group. ^a^*P* = 0.044 for overall group differences across time; *P* = 0.067 for overall time effect. ^b^*P* = 0.007 (adj *P* = 0.028) for Combined *vs* Control groups, after nature-based program (Assmt-1). ^c^*P* = 0.009 (adj *P* = 0.036) for Combined *vs* Control groups, after nature-based and audio-based mindfulness programs (Assmt-2). (B) NIH-HEALS scores over time in the Intervention (Combined or Nature) and Control groups. ^a^*P* = 0.019 for overall group differences across time; *P* = 0.10 for overall time effect. ^b^*P* = 0.009 (adj *P* = 0.036) for Intervention *vs* Control groups, after nature-based program (Assmt-1). ^c^*P* = 0.005 (adj *P* = 0.020) for Intervention *vs* Control groups, after nature-based and audio-based mindfulness programs (Assmt-2). ^d^In the Intervention group, baseline (Assmt-0) *vs* each of Assmt-1 (corresponding to after nature-based program; adj *P* = 0.006), Assmt-2 (corresponding to after nature-based and audio-based mindfulness; adj *P* < 0.001), and Assmt-3 (corresponding to after the 2-month follow-up; adj *P* = 0.048); no differences were noted over time in the control group.

The mean baseline NIH-HEALS score was 123.1 ± 19.4 in the Combined, 118.1 ± 17.1 in the Nature, and 114.3 ± 17.8 in the Control groups (Table 2). At the primary endpoint (Assmt 2), the mean NIH-HEALS score increased to 129.7 ± 19.7 in the Combined and 126.8 ± 21.3 in the Nature groups. The mean score in the Control group, at the primary endpoint, was 112.0 ± 15.3. At follow-up, the secondary time point for the intervention groups, the NIH-HEALS increased in the Combined group (131.3 ± 21.2) but decreased in the Nature group (120.4 ± 23.9) (Table 2). Overall global tests of significance showed a weak group effect (*p* = 0.044) and no time effect (*p* = 0.067) (Fig. 4A). However, aggregating data from the Combined and Nature groups into a single intervention arm yielded a substantial improvement in NIH-HEALS score when compared to the Control group (*p* = 0.019) (Fig. 4B).

## Discussion

The present study reports the effects of a nature activity and a combined nature and mindfulness intervention on the stress and wellbeing of COVID-19 HCWs. The results demonstrated that engaging in nature-based and mindfulness-based activities did not significantly impact the stress levels as was hypothesized and assessed by the PSS, the primary outcome measure. The results, however, demonstrated that nature activity and nature combined with mindfulness can improve psychosocial-spiritual wellbeing as assessed by the NIH HEALs, a secondary outcome measure. The Combined group had greater improvement in wellbeing when compared to the Nature and the Control groups. Similarly, the Nature group had greater improvement in wellbeing compared to the Control group. Finally, at follow-up, the Combined group maintained improvements in wellbeing when compared to the Nature group. There were no notable changes in stress levels among the groups.

Mean PSS scores among our groups ranged from 21.2–23.2 at baseline and 20.9–21.1 at follow-up. These score ranges are consistent with moderate stress levels. PSS did not detect change neither between nor within the groups. Several factors might explain this result. The low statistical power of the study likely hindered our ability to detect change. Additionally, changes in PSS scores are typically small with 0.25−0.50 SD changes indicating meaningful effect. Given our small sample size, these small changes may have been particularly challenging to capture.

While a great deal of research report positive outcomes in mindfulness and nature exposure studies (Corazon et al., 2019; Green & Kinchen, 2021; Khoury et al., 2015; Van Den Berg & Custers, 2011; Van Dijk et al., 2017; Yang et al., 2023), the effectiveness of nature-based and mindfulness-based interventions on stress-related issues have also produced mixed results including or no improvements (Ghawadra et al., 2020; Goldhagen et al., 2015; Mygind et al., 2019; Tillmann et al., 2018). Differences in study design may account for these discrepancies. For instance, dose–response in both nature and mindfulness interventions are potentially quite important and such information are gradually emerging. Some studies cite that the most effective nature interventions typically span 8 to 12 weeks, with an optimal weekly dose of 20–90 min (Coventry et al., 2021). Similarly, standard mindfulness-based therapies often last 8 weeks, totaling in about 30-hours in MBSR^TR^. The current interventions were briefer with 3–5 days of nature intervention, and a total of 3.5 h of mindfulness intervention. Although brief mindfulness interventions have been reported, they generally exceed four hours; for example, a 7.5-hour program effectively reduced stress in HCWs (Ameli et al., 2020). Interestingly, a study using mobile mindfulness applications for 10-minutes daily over 10 days significantly improved depressive symptoms and mindfulness (Flett et al., 2019).

In the present study, overall wellbeing was assessed using the NIH-HEALS, a measure of psychosocial-spiritual wellbeing. Results show that NIH-HEALS scores increased over time for both the Nature and the Combined groups. While these improvements were not statistically substantiated in between-group comparisons, aggregating the data from the intervention groups revealed a meaningful improvement in wellbeing relative to the control. Moreover, follow-up analyses showed that only the Combined group maintained its improvement two-months post-intervention. These findings indicate that nature programs improve wellbeing and that adding a mindfulness program may sustain the benefits overtime. As mentioned before, the study was not sufficiently powered to produce definitive results.

Our results are consistent with previous research showing that nature-based interventions improve overall wellbeing (Shanahan et al., 2016; Yang et al., 2023). These interventions, which include activities like therapeutic gardening and exposure to green spaces, have been shown to enhance mood, quality of life, and to increase overall emotional and psychological health (Annerstedt & Wahrborg, 2011; Petitt, Rolander & Johnsson, 2023; Van Den Berg & Custers, 2011). In the context of workplace wellbeing, incorporating nature activities could lead to better job and life satisfaction, and cognitive engagement in daily work activities (Gritzka et al., 2020).

Notably, however, the effects of nature-based interventions can be short-lasting (Kotera, Richardson & Sheffield, 2022). Our results indicate that mindfulness-based interventions can be combined with nature interventions to sustain positive psychosocial-spiritual wellbeing changes for at least two months. This is in line with previous research findings. Mindfulness interventions have been shown to promote psychosocial wellbeing and improve resilience in healthcare professionals (Lomas et al., 2019; Shiri, Nikunlaakso & Laitinen, 2023; Van Dijk et al., 2017). Recent evidence even suggests similar benefits from brief mindfulness programs as well as app-based digital interventions (Ameli et al., 2020; Taylor et al., 2022). Mindfulness interventions, including virtual guided meditations, such as mindful breathing exercises, and body scans, provide flexibility and accessibility. Short-term, audio, online, and virtual mindfulness interventions can be particularly viable methods to enhance wellbeing in HCWs with busy and or inflexible schedules.

There are several limitations to consider when interpreting the results. The study was underpowered and therefore the potential impact of the interventions was not fully captured. The small sample size and demographic profile of the participants, predominantly female, non-Hispanic white Christian nurses, may limit the generalizability of our findings to other groups and/or healthcare professionals. Nonetheless, the group adequately represents the healthcare workforce in the USA, with nurses being the predominant profession and women comprising over 87% of nursing workforce (U. S. Bureau of Labor Statistics, 2023). Our study spanned over two years, during which the profile and impact of COVID-19 evolved and changed. This could have influenced the participants stress levels as well as their motivation for participation in the study.

Finally, the drop-out rate in the intervention groups was high, at around 40%. While this is significant, attrition rates have been cited as high as 38% for face-to-face mindfulness interventions, with a mean of 19.1% (Lam, Kirvin-Quamme & Goldberg, 2022; Strauss et al., 2014). Attrition rates in nature-based interventions is not well documented, although a recent study reported a rate of 29% (Vermeesch et al., 2022). Among online mindfulness interventions, attrition rates can be seen as high as 59% (Nadler, Carswell & Minda, 2020). High attrition rates in online mindfulness interventions are largely attributed to the voluntary nature of participation and the lack of face-to-face instruction, which often leads to lower engagement compared to in-person programs.

In sum we did not detect change in stress levels, but nature and mindfulness interventions improved wellbeing. Future studies with larger sample size are needed to further elucidate the effectiveness of mindfulness interventions combined with nature activities, to improve and sustain the impact of nature related interventions.

## Supplemental Information

10.7717/peerj.19109/supp-1 Supplemental Information 1 Raw data and Codebook All participants, their demographic information and their scores on both instruments. These data were used for statistical analysis to compare the different intervention groups.

10.7717/peerj.19109/supp-2 Supplemental Information 2 Additional endpoints at study intervals in a cohort of frontline COVID-19 healthcare workers randomized to nature-based and audio-based mindfulness interventions, and controls Randomized groups were combined Nature-Mindfulness (Combined), Nature-only (Nature), and Control groups. Sample sizes are listed where data were missing. Assessment 3 was not applicable to the Control group.

10.7717/peerj.19109/supp-3 Supplemental Information 3 CONSORT checklist

10.7717/peerj.19109/supp-4 Supplemental Information 4 Trial protocol

## Additional Information and Declarations

### Data Availability

The following information was supplied regarding data availability: The raw demographic data of the participants and the scores from the HEALS and PSS instruments are available in the Supplementary File.
