# Supplementary material for: Effects of a combined nature-based and audio-based virtual mindfulness intervention on stress and wellbeing of COVID-19 healthcare workers: a randomized controlled trial
Source: PeerJ. 2025 May 23;13:e19109. doi: 10.7717/peerj.19109 (PMC12105617; doi:10.7717/peerj.19109)
Supplement: Supplemental Information 2 — Randomized groups were combined Nature-Mindfulness (Combined), Nature-only (Nature), and Control groups. Sample sizes are listed where data were missing. Assessment 3 was not applicable to the Control group. [file peerj-13-19109-s002.docx]

**Supplemental Table 1:**

**Additional endpoints at study intervals in a cohort of frontline COVID-19 healthcare workers randomized to nature-based and audio-based mindfulness interventions, and controls.**

Randomized groups were combined Nature-Mindfulness (Combined), Nature-only (Nature), and Control groups. Sample sizes are listed where data were missing. Assessment 3 was not applicable to the Control group.

|  | **Combined**  **(n=16)** | **Nature**  **(n=19)** | **Control**  **(n=18)** |
| --- | --- | --- | --- |
| **ISI**, total score, mean ±SD  Assmt-0  Assmt-1  Assmt-2**^*^**  Assmt-3 | 7.6 ±5.4  6.4 ±4.1  5.8 ±4.4  6.6 ±3.5 (n=14) | 12.1 ±4.6  11.2 ±4.3  9.7 ±4.4  7.6 ±4.3 (n=17) | 11.1 ±5.4  10.8 ±4.3  9.8 ±4.6  (n/a) |
| **MBI-2**, total score, mean ±SD  Assmt-0  Assmt-1  Assmt-2**^*^**  Assmt-3 | 5.5 ±2.3  5.4 ±2.9  4.5 ±2.2  4.5 ±2.5 (n=14) | 7.6 ±2.6  7.7 ±2.8  6.5 ±2.6  7.2 ±2.3 (n=17) | 7.7 ±2.9  7.2 ±2.6  7.4 ±2.8  (n/a) |
| **PCL-5**, total score, mean ±SD  Assmt-0  Assmt-1  Assmt-2**^*^**  Assmt-3 | 14.1 ±11.2  14.8 ±11.7  9.6 ±8.7  11.1 ±8.6 (n=14) | 24.9 ±13.8  25.2 ±12.3  18.1 ±9.7  20.2 ±11.8 (n=17) | 25.2 ±9.5  21.3 ±9.0  20.3 ±10.8  (n/a) |
| **HADS**, total score, mean ±SD  **Anxiety**  Assmt-0  Assmt-1  Assmt-2**^*^**  Assmt-3  **Depression**  Assmt-0  Assmt-1  Assmt-2**^*^**  Assmt-3 | 6.4 ±3.5  4.7 ±2.9  4.7 ±3.6  5.0 ±3.1 (n=13)  4.4 ±3.8  2.4 ±2.7  2.9 ±3.3  3.1 ±3.6 (n=13) | 8.5 ±4.1  7.6 ±3.7  8.1 ±3.3  9.1 ±3.3 (n=17)  5.9 ±2.7  4.9 ±3.7  5.4 ±4.0  5.7 ±3.8 (n=17) | 11.1 ±2.7  9.4 ±2.5  9.6 ±1.8  (n/a)  7.3 ±2.3  7.0 ±3.2  7.1 ±3.2  (n/a) |
| **MAAS**, total score, mean ±SD  Assmt-0  Assmt-1  Assmt-2**^*^**  Assmt-3 | 4.0 ±1.4  4.2 ±1.6  4.3 ±0.9  3.6 ±1.3 (n=13) | 2.9 ±1.3  3.7 ±1.4  3.5 ±1.6  3.2 ±1.4 (n=17) | 2.9 ±1.6  3.2 ±1.6  3.4 ±1.6  (n/a) |
| **GSF**, total score, mean ±SD  Assmt-0  Assmt-1  Assmt-2**^*^**  Assmt-3 | 22.5 ±4.0  22.8 ±2.4  22.4 ±3.5  24.0 ±3.6 (n=13) | 20.2 ±4.4  20.7 ±4.6  21.3 ±4.6  20.4 ±5.8 (n=17) | 20.9 ±3.0  19.8 ±3.2  20.1±3.2  (n/a) |

Abbreviations: Assmt- Assessment; ISI, Insomnia Severity Index; MBI-2, Maslach Burnout Inventory; PCL-5, Posttraumatic Stress Disorder Checklist for DSM-5; HADS, Hospital Anxiety and Depression Scale; MAAS, Mindful Attention Awareness Scale; GSF, General Self-Efficacy Scale.

*Primary outcome.

**The Insomnia Severity Index (ISI)** is a brief screening tool with 7 items and is scored on a 4-point Likert scale. It is designed to identify insomnia, and measures quality of sleep. This validated tool demonstrated an internal consistency of α=0.74.

**The Maslach Burnout Inventory (MBI-2 item)** has 22 items and evaluates self-reported burnout. The 2-item scale has demonstrated strong validity, reliability, and high correlation with the full MBI, and is separately scored on a 7-point Likert scale. One item assesses emotional exhaustion, and a second item assesses depersonalization.

**The Posttraumatic Stress Disorder Checklist for DSM-5 (PCL-5)** is a widely used 20-item self-report measure and is scored on a 5-point Likert scale. It assesses the severity of 20 *DSM5* symptoms of PTSD.  PLC-5 has high reliability (Cronbach’s alpha =0.94), and validity (convergent r =0.74 to 0.85).

**The Hospital Anxiety and Depression Scale (HADS)** is a popular self-report screening tool for clinical and research purposes. It consists of 14 items and is scored on a 4-point Likert scale. It contains two 7-item scales: one for anxiety and one for depression, both with a score range of 0 to 21. It has high validity and reliability. Cronbach’s alpha values for HADS Anxiety and HADS Depression are 0.92 and 0.88, respectively.

**The Mindful Attention Awareness Scale (MAAS)-state version** is a 5-item self-report scale on a 7-point Likert scale. This measure assesses the short-term expression of mindfulness. Cronbach’s alpha ranges between 0.89 and 0.93.

**The General Self-Efficacy Scale (GSF)** is a 10-item self-report questionnaire and is scored on a 4-point Likert scale. It assesses a general sense of perceived self-efficacy by predicting coping and adaptation levels after stressful life events. This scale has high internal consistency, reliability, and predictive validity with Cronbach’s alpha = 0.76 to 0.90.
