# Supplementary material for: Effects of a combined nature-based and audio-based virtual mindfulness intervention on stress and wellbeing of COVID-19 healthcare workers: a randomized controlled trial
Source: PeerJ. 2025 May 23;13:e19109. doi: 10.7717/peerj.19109 (PMC12105617; doi:10.7717/peerj.19109)
Supplement: Supplemental Information 4 [file peerj-13-19109-s004.pdf]

Trial protocol

Effects of a combined Nature-Based and Audio-Based Virtual Mindfulness intervention on stress and wellbeing of COVID-19 healthcare workers: a Randomized Controlled Trial

Principal Investigator: Ann Berger, MD

RESEARCH PROPOSAL

Study Rationale

Healthcare workers caring for patients with COVID-19 experience significant stress and trauma exposure with increased risk for psycho-social-spiritual distress. Social distancing has further limited services available to address mental health needs. Feasible and effective interventions are needed to reduce stress and increase wellbeing and resilience in this population. This randomized controlled trial aims to investigate the feasibility and efficacy of a combined nature and mindfulness intervention by sequencing a nature program followed by an audio-based mindfulness intervention on primary outcomes of stress, as well as secondary outcomes of sleep, burnout, posttraumatic stress, anxiety, depression, mindfulness, self-efficacy, and psycho-social-spiritual healing. To our knowledge no such augmentation has been previously studied.

Background

The COVID-19 pandemic has imposed a tremendous amount of psychosocial distress on the healthcare workforce. Working under high levels of stress can increase the risk for developing a myriad of mental health symptoms.[1] A large recent cross-sectional study in the U.S. with data from 657 clinicians at New York-Presbyterian/ Columbia University and other collaborators assessing nurses and advanced practice providers, confirms a significantly high prevalence of psychological symptoms such as acute stress, anxiety, depression, sleep disturbances, and feelings of loneliness.[2] Another study conducted in China had shown similar symptoms in frontline healthcare workers experiencing elevated levels of distress, anxiety, and depression.[3] The nature of the work can be traumatic, as it may involve witnessing the death of patients and colleagues, and the imminent fear of exposing self and family to a fatal virus.[4,5] In addition, they are at increased risk for developing trauma-related disorders, a trajectory consistent with some providers who worked during the previous viral outbreaks.[6] Moreover, the unprecedented demands of caring for patients can compromise sleep quality and lead to insomnia.[7,8] Not only is insomnia highly comorbid with mental health conditions, but multiple longitudinal studies indicate that sleep disturbances contribute to the prediction of new-onset posttraumatic stress, anxiety, and depressive disorders,[9-14] with meta-analytic findings reporting that sleep disturbance yields a three-fold increased risk of anxiety and depression.[15] Chronic stress and inadequate sleep may culminate in burnout, which is characterized by emotional exhaustion, depersonalization, and a sense of low personal accomplishment.[16] Burnout is consistent with decreased resilience, poor psychological recovery, which can exacerbate distress.[17]

Social distancing can limit availability of mental health services that could meet the needs of frontline healthcare workers. Innovative interventions that are feasible and effective are needed to

reduce stress and increase resilience in this population. Studies have shown that complementary and ancillary programs can increase resilience and wellbeing. Among ancillary services many involve outdoor activities[18], nature adventures[19,20], and spending time in nature.[21,22] Nature exposure can enhance well-being with the potential to reduce adverse effects of stressors.[21] Research on First Descents' programs, which primarily include nature-based activities, demonstrated that young adult cancer survivors who participated in the program had decreased stress, fewer symptoms of depression and alienation, and improved self-efficacy.[23,24] However, individuals who participated in the program a second time were not better off psychologically than those who participated only once. Writers argued that individuals signed up for a second time because the benefits of the first program could have faded.[23] This suggests that the benefits derived from this type of outdoor programs can be short-lived and may require additional therapeutic components to strengthen and prolong their efficacy.[25]

Mindfulness interventions are evidence-based and are a strong candidate to combine with nature programs for added benefit. Mindfulness is defined as "paying attention on purpose, in the present moment, and non-judgmentally to the unfolding of experience moment by moment".[26] Meta-analyses have shown that mindfulness-based interventions reduce stress and burnout,[27] improve sleep quality,[28] alleviate anxiety and depression,[29,30] and improve and increase resilience.[31] Audio-based mindfulness applications have gained popularity and many studies support their effectiveness.[32,33] Since social distancing has rendered face-to-face mindfulness-based interventions infeasible, audio-based mindfulness applications may be a viable alternative. Indeed, the portability of these applications reduce the burden of accessing mindfulness-based interventions. These mindfulness applications have demonstrated durable positive effects on wellbeing. For instance, a randomized controlled trial found that experimental participants (n=191) compared to controls (n=186), endorsed large decreases in psychiatric symptoms and moderate improvements in psychological, social, and quality of life measures after using an audio-based smartphone mindfulness application for five weeks, and these gains were maintained for at least three months.[34] Another study using a relatively brief mobile mindfulness application for 10 minutes a day for 10 days[35] reported significant improvements in depressive symptoms and mindfulness in the experimental group. Other studies have also supported the effectiveness of brief mindfulness applications.[36]

Purpose of the Proposed Study

Stress-related symptoms in COVID-19 healthcare workers is an important public health issue that requires attention. A previous study conducted in our lab has shown that a brief mindfulness intervention decreases stress in healthcare providers.[37] Another study from our lab confirms the positive impact of nature on stress reduction.[38] We also found that adding a mindfulness component to the initial nature experience instructions, enhanced and enriched participants' positive experiences and contributed to a present moment orientation and increased relaxation.[38]

In the present study we propose to combine a nature program with an audio-based mindfulness intervention. Our goal is to investigate the feasibility and effectiveness of the combined sequenced interventions to decrease stress in COVID-19 healthcare workers. In addition, we will investigate whether there is a decrease in sleep disturbance, burnout, posttraumatic stress, anxiety, depression, as well as improvements in resilience assessed by increased mindfulness, self-efficacy, and psycho-social-spiritual healing. Ninety COVID-19 healthcare workers will be randomized into one of three

groups: Nature+Mindfulness (*n*=30), Nature only (*n*=30), and a waitlist control (*n*=30). We hypothesize that the Nature+Mindfulness group will have greater reductions in stress than the other two groups. In addition, the Nature+Mindfulness group will have greater reductions in sleep disturbance, burnout, posttraumatic stress, anxiety, depression, as well as greater improvements in resilience assessed by increased mindfulness, self-efficacy, and psycho-social-spiritual healing compared with the other two groups. Moreover, we hypothesize that at 10-week follow-up the Nature+Mindfulness group will demonstrate a greater maintenance of positive gains compared with the Nature only group.

Specific Aims

Primary Aim:

1.) To investigate the feasibility and effectiveness of a Nature+Mindfulness stress reduction program.

> Hypothesis 1: The Nature+Mindfulness group will have greater reductions in stress indicated by decreased Perceived Stress Scale (PSS) compared to the Nature only and waitlist control groups.
> Hypothesis 2: The Nature only group will have greater reduction in stress as indicated by decreased PSS compared to the waitlist control group.
> Hypothesis 3: Assessment of response rate, attendance rate, participant feedback and adverse events will demonstrate that Nature+Mindfulness intervention is feasible.

Secondary Aims:

2.) To investigate the effect of Nature+Mindfulness intervention on psycho-social-spiritual healing.

3.) To investigate intervention gains at 10-week follow-up in the Nature+Mindfulness and Nature only groups.

> Hypothesis 1: The Nature+Mindfulness group will better retain stress reduction and other positive gains compared to the Nature only group.

Research Strategy

Study Population and Procedures. The study will recruit 90 (male and female age 18 and up) active COVID-19 healthcare workers through First Descents organization.

Study Design. Ninety COVID-19 healthcare workers will be randomized into one of three groups: Nature+Mindfulness (*n*=30), Nature only (*n*=30), and a waitlist control (*n*=30). The randomization scheme will be based on block sizes of six, with 2:2:2 random assignments into each group. De-identified data will be collected from eligible participants who will directly enter their information and responses into a database management system. The Nature+Mindfulness group will undergo both the nature program and mindfulness intervention. The Nature only group will participate only in the nature program. The waitlist control group will participate in the nature program at a later date after study completion. Figure 1 depicts the study design, groups, and study intervals.

Experimental Plan and Interventions

Nature Program. Nature programs are found to be therapeutic, reduce stress, increase relaxation, and improve well-being.[39,40] First Descents has provided nature adventure programs to young adults with cancer and other serious illness with positive results.[23] A nature program for COVID-19 frontline healthcare workers has been offered by First Descents since August 2020. The program is three days long and includes rock climbing and hiking in a nature-rich environment.

Mindfulness Intervention. Mindfulness is established as an effective stress-reduction intervention.[41,42] Our mindfulness intervention will include an audio-based intervention to be used for 10 days after the nature program is completed (See Research Methods, Figure 1). Audio-based mindfulness applications have gained popularity and there is significant literature that supports their effectiveness.[43] Various smartphone applications have been studied for their impact on medical personnel with positive results.[44,45] There are also reports that indicate their positive impact on mood, stress, mindfulness, and well-being even with short duration of practice.[46-48] Audio-based mindfulness applications provide a safe, and portable alternative to regular group-based, face-to-face interventions that are typically 30 hours in length.

Measures. Outcomes will be assessed with validated measures and take approximately 35-40 minutes to complete. These will be completed up to four times per participant.

Primary Outcome: The Perceived Stress Scale (PSS) is used for the primary outcome measure. It is a 10-item self-report scale and is scored on a 0-4 scale ranging from never to very often. This is a widely used and validated measure with Cronbach's alpha ranging between 0.75 and 0.91.[49,50]

Secondary Outcome: The National Institutes of Health – Healing Experience of All Life Stressors (**NIH-HEALS)** is a 35-item self-report questionnaire on a 5-point Likert scale developed by the NIH Clinical Center Pain and Palliative Care Service. NIH-HEALS is a measure of healing that assesses an individual's psycho-social-spiritual mechanisms for coping during life's difficult situations and/or life-limiting challenges. There is strong evidence for its validity and reliability (Cronbach's alpha = 0.89, split-half reliability=0.95).[62,63]

Participation Flow. After consenting to take part in the research, participants will complete a demographic questionnaire and the first set of assessments through REDCap one week to 2 days prior to the Nature program participation. REDCap prompts will be emailed throughout the study. The estimated time to complete the questionnaire and assessments is about 1 hour. One day after the completion of the nature program, those in the Nature+Mindfulness group and the Nature only group will complete the second set of assessments. The control subjects will also complete the second assessment in a corresponding time frame. These will be completed via REDCap and will take 35-45 minutes. After the second assessment, those in the Nature+Mindfulness group will receive the mindfulness program manual through email and mail, which describes mindfulness and provides information and references. Participants in the Nature+Mindfulness group will also receive daily audio recordings of mindfulness through REDCap for 10 days. Recordings will vary in length from 20-25 minutes. During these 10 days, participants will also receive REDCap prompts to confirm if they have

listened and completed daily mindfulness practice. These prompts can be completed in less than 2 minutes. After the 10 days, all participants will complete the third assessment through REDCap, estimated to take 35-45 minutes. All participants except those in the control group will then receive weekly reminders to stay in touch and see if they are engaged in self-care activities via a short survey taking less than 2 minutes. After eight weeks, these same participants (Nature+Mindfulness and Nature only groups) will complete the fourth and final assessment through REDCap, estimated to take 35-45 minutes. This will complete their participation in the study.

Statistical Plan. The primary and secondary outcome data will be collected from all three groups: Nature+Mindfulness, Nature only, and waitlist control at three time intervals (**Figure 1**). In addition, all groups will complete a demographic questionnaire at the time of the first assessment. The Nature+Mindfulness (experimental) group will complete study measures at baseline (interval 0), within one to two days after completing the nature program (interval 1), and within one to two days after completing the audio mindfulness intervention (interval 2). During the 10-day mindfulness intervention, participants will receive daily reminders from REDCap regarding their practice, which also includes the rating of their stress level. These data will be for descriptive purposes only. The Nature only group will complete study measures at baseline (interval 0), within one to two days after completing the nature program (interval 1), and again 11-12 days after the nature program (interval 2, corresponding to the post-mindfulness interval of the experimental group). The waitlist control group will complete the study measures at baseline (interval 0), one week after baseline (interval 1), and again 11-12 days later (interval 2), corresponding to the experimental group's timeline. The Nature+Mindfulness and Nature only groups will have one additional follow-up at 10-weeks (interval 3) when they will complete the measures one last time. The weekly check-in data collected during the follow-up period will be used to describe self-care behaviors by the participants in these groups. These data will be compared between the two groups (Nature+Mindfulness vs Nature only). These two groups will also receive a Program Evaluation form designed based on their respective experiences.

The primary outcome of the study will be stress level measured by PSS compared among the three groups, at the third time interval that corresponds to the completion of the mindfulness intervention by the experimental group (**Figure 1**). Data will be analyzed using Generalized Linear Mixed Models for repeated measures, taking into account the baseline levels and within-group comparisons across time. Mixed models have several advantages for such a design: 1) they can be used for modeling non-normal response variables and for modeling discrete responses alike, 2) they account for within subject correlations in repeated measures, 3) utilize all available data even in the presence of missing components, and 4) have greater power by utilizing all available data for more appropriate inferences. The models will include other covariates, such as participant age and sex, and other potential confounders. A demographics questionnaire will collect information about the participants that will also include profession, and level and type of exposure to COVID-19. Post-hoc analyses will be carried out for specific pairwise comparisons (e.g., Nature+Mindfulness vs Nature only, Nature only vs waitlist control), adjusting for multiple comparisons using the Stepdown Bonferroni method. This method is appropriate for the analysis of the primary outcome (as described), as well as for the secondary outcomes. Selection bias is a limitation in this study, and therefore results will be generalizable to a target population similar to those who participate in this study.

Sample Size: A detection of a 4.0 difference in the PSS score, the primary outcome, was determined to be a meaningful change. Sample size estimation was based on a two-sided t-test, with unequal variances, power at 0.85, delta of 4.0, and standard deviations of 4.0. Alpha was set at 0.0167 to account for the comparison of three groups. This yielded a sample size of 26 per group. Considering a 15% dropout or lost-to-follow-up rate, a total of 30 participants per group would be needed. Thus, the study will enroll 30 participants in the Nature+Mindfulness, 30 in Nature only, and 30 in the waitlist control groups, for a total of 90 participants.

1. Recruitment & Screening

The study will recruit active COVID-19 healthcare workers with assistance from First Descents (FD) organization. First Descents, founded in 2001, is a non-profit 501(c)(3) organization which provides free outdoor adventure activities to young adults impacted by cancer and other serious health conditions. First Descents, which is based in Colorado and has provided nature programs across the United States for the past 20 years, is currently offering nature-based programs for COVID-19 healthcare workers. Interested applicants in the nature program will receive an email from FD introducing them to the NIH research project. When the subject agrees, then FD will share the subject's contact information (first name, last name, phone number, and email address) with our team. Interested applicants will be screened by phone. If qualified, they will receive a unique number to sign on to the database program (REDCap), read about the study group to which they have been assigned, read consent document, and if interested to participate they will continue with a demographic questionnaire and fill out the first set of assessments (See below Primary and Secondary Outcome Measures). To enroll in the study, participants must meet the following criteria:

FD will not have access to the NIH data.

Inclusion & Exclusion Criteria include the following

Study Inclusion Criteria:
1. Age 18 and older;
2. Is a healthcare worker having provided care to COVID-19 patients;
3. Has internet and device access to fill out questionnaires;
4. Is fluent in English;
5. Is able to provide their own consent.

Study Exclusion Criteria:
1. Is experiencing an acute psychiatric condition;
2. Has hearing impairments that are not modified with aids or devices.

Data Collection

Data will be collected de-identified. Participants will be given a random unique number to sign on to the database (REDCap) where they input their data directly. Names, phone numbers, and emails will be collected for screening purposes. Data and linking codes will be kept at the NIH and will not be entered into the database. Only the unique subject number will be linked to their groupings and data collection. The screening data will not be part of the study data and will only be used to determine eligibility. The Principal Investigator will oversee the storage and management of data. Database manager (Frank Velez) will have access to the codes but no access to the screening information names, phone numbers, or emails.

2. **Consent Process:** The consent process has 3 stages
    1. An initial letter by FD offering the opportunity to partake in research. If prospective subject expresses interest and agrees, FD will provide the NIH with contact information (name, email, and phone as well as providing NIH contact information to the subjects).

    2. Interested subjects will be screened by the phone and if eligible will be provided with a unique number to sign on to the database (REDCap). They will be assigned to one of the 3 study groups.

    3. After reading specific and detailed description of the group to which they have been assigned, then they will read an additional consent document. If the subject agrees to the specific research details, and reads the consent document and agrees to participate, then they will be included in the study. From this point on, they will receive prompts from REDCap which guides the participants through the research steps based on their group assignment. If the participant does not consent to participation at this point, their access to REDCap will be terminated.

    4. Justification for waiver of documentation of informed consent:

    That the research presents no more than minimal risk of harm to subjects and involves no procedures for which written consent is normally required outside of the research context.
3. **Research Methods**

See study design pg. 5

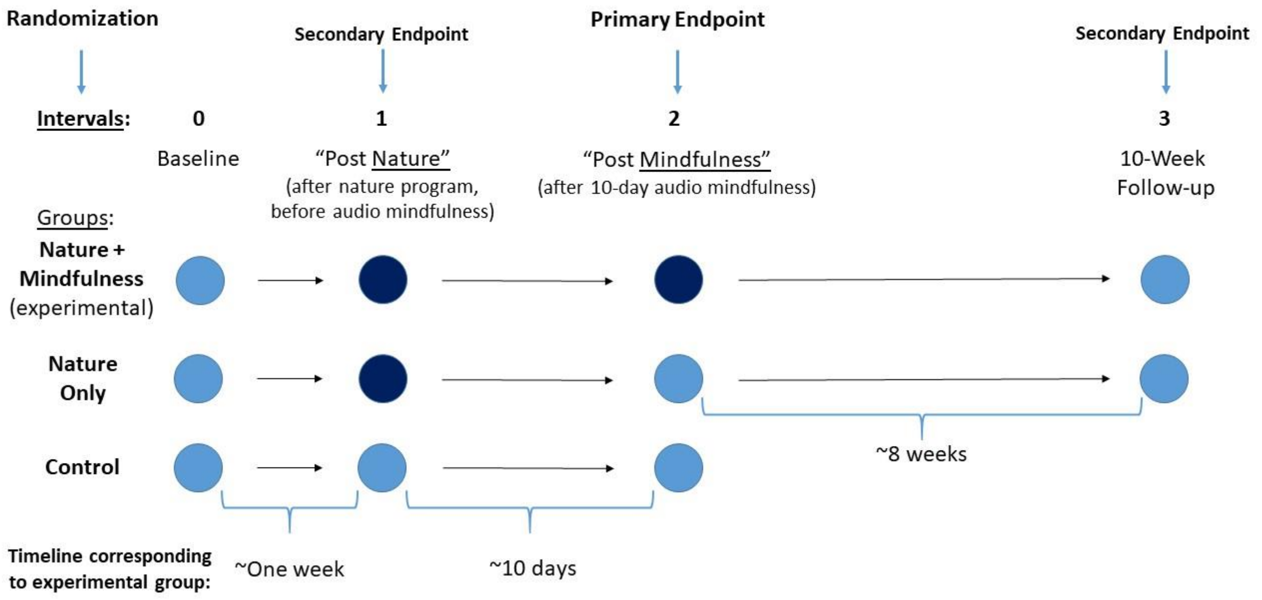

Figure 1. Study design and flow diagram of study groups and intervals. Dark circles represent applicable interventions. The timeline/intervals correspond to the experimental group.

Experimental Plan and Interventions

Nature Program: Nature programs are found to be therapeutic, reduce stress, increase relaxation, and improve well-being.[39,40] First Descents has provided nature adventure programs to young adults with cancer and other serious illness with positive results.[23] A nature program for COVID-19 frontline healthcare workers has been offered by First Descents since August 2020. This one-time program is three days long and includes rock climbing, hiking, and kayaking in a nature-rich environment. The program is in-person, and will be offered on different dates at the location in which the participants live.

Mindfulness Intervention: Mindfulness is established as an effective stress-reduction intervention.[41,42] Our mindfulness intervention will include audio-based recordings to be used for 10 days after the nature program is completed (Figure 1). Audio-based mindfulness applications have gained popularity and there is significant literature that supports their effectiveness.[43] Various audio-based applications have been studied for their impact on medical personnel with positive results.[44,45] There are also reports that indicate their positive impact on mood, stress, mindfulness, and well-being even with short duration of practice. Audio-based applications provide a safe, and portable alternative to regular group-based, face-to-face interventions that are typically 30 hours in length. We have recently demonstrated the efficacy of a relatively short mindfulness-based self-care program. Each participant will be mailed a hard copy and emailed the PDF of the manual describing the mindfulness intervention. The 10-day audio-based recordings will be sent to the participants through REDCap. Each day, the participants will receive a reminder through REDCap and will complete a brief questionnaire about their practice. On average, the mindfulness audio-recordings are 20-25 minutes long, for a once-a-day practice. The mindfulness intervention is developed for this research through the Clinical Center Communications Office.

Participation Flow. After consenting to take part in the research, participants will complete a demographic questionnaire and the first set of assessments through REDCap one week to 2 days prior to the Nature program participation. REDCap prompts will be emailed throughout the study. The estimated time to complete the questionnaire and assessments is about 1 hour. One day after the completion of the nature program, those in the Nature+Mindfulness group and the Nature only group will complete the second set of assessments. The control subjects will also complete the second assessment in a corresponding time frame. These will be completed via REDCap and will take 35-45 minutes. After the second assessment, those in the Nature+Mindfulness group will receive the mindfulness program manual through email and mail, which describes mindfulness and provides information and references. Participants in the Nature+Mindfulness group will also receive daily audio recordings of mindfulness through REDCap for 10 days. Recordings will vary in length from 20-25 minutes. During these 10 days, participants will also receive REDCap prompts to confirm if they have listened and completed daily mindfulness practice. These prompts can be completed in less than 2 minutes. After the 10 days, all participants will complete the third assessment through REDCap, estimated to take 35-45 minutes. All participants except those in the control group will then receive weekly reminders to stay in touch and see if they are engaged in self-care activities via a short survey taking less than 2 minutes. After eight weeks, these same participants (Nature+Mindfulness and Nature only groups) will complete the fourth and final assessment through REDCap, estimated to take 35-45 minutes. This will complete their participation in the study.

Measures: Outcomes will be assessed with validated measures and take approximately 35-40 minutes to complete. These will be completed up to four times per participant.

Sample Size: A detection of a 4.0 difference in the PSS score, the primary outcome, was determined to be a meaningful change. Sample size estimation was based on a two-sided t-test, with unequal variances, power at 0.85, delta of 4.0, and standard deviations of 4.0. Alpha was set at 0.0167 to account for the comparison of three groups. This yielded a sample size of 26 per group. Considering a 15% dropout or lost-to-follow-up rate, a total of 30 participants per group would be needed. Thus, the study will enroll 30 participants in the Nature+Mindfulness, 30 in Nature only, and 30 in the waitlist control groups, for a total of 90 participants.

4. **Compensation:** there will be no compensation for study participation.

5. **RISKS and Benefits**:

Risks: We are not aware of any actual harm that can come from the study. The risks associated with this study are minimal. Minimal risk means that the **probability and magnitude of harm or discomfort anticipated in the research are not greater** than those ordinarily encountered in daily life and activities.

- <u>Self-report questionnaires:</u> There is minimal medical risk in completing the self-report questionnaires. Some of the questions may elicit discomfort or anxiousness. Participants may refuse to answer any question or to stop answering the questionnaires at any time and for any reason.

- Storage/sharing of data and samples: There is minimal risk that data could be identified. To minimize this risk, data will be collected de-identified. Data and linking code will be kept at the NIH. FD will not have access to the NIH data.
- Mindfulness intervention: We are not aware of any harm from brief mindfulness interventions.

Benefits: activities and practices selected for the study are known to reduce stress. However, we do not guarantee or promise that participants will receive any benefits from the study.

6. **Data Analysis Plan:** The primary and secondary outcome data will be collected from all three groups: Nature+Mindfulness, Nature only, and from the waitlist control at three-time intervals (**Figure 1**). In addition, all groups will complete a demographic questionnaire at the time of the first assessment. The Nature+Mindfulness (experimental) group will complete study measures at baseline (interval 0), within one to two days after completing the nature program (interval 1), and within one to two days after completing the audio-based mindfulness intervention (interval 2). During the 10-day mindfulness intervention, participants will receive daily reminders from REDCap regarding their practice, which also includes the rating of their stress level. These data will be for descriptive purposes only. The Nature only group will complete study measures at baseline (interval 0), within one to two days after completing the nature program (interval 1), and again 11-12 days after the nature program (interval 2, corresponding to the post-mindfulness interval of the experimental group). The waitlist control group will complete the study measures at baseline (interval 0), one week after baseline (interval 1), and again 11-12 days later (interval 2), corresponding to the experimental group's timeline. The Nature+Mindfulness and Nature only groups will have one additional follow-up at 10-weeks (interval 3) when they will complete the measures one last time. The weekly check-in data collected during the follow-up period will be used to describe self-care behaviors by the participants in these groups. These data will be compared between the two groups (Nature+Mindfulness vs Nature only). These two groups will also receive a Program Evaluation form designed based on their respective experiences. Nature+Mindfulness will also receive a Manual in written form that provides additional information to the subjects about mindfulness.

The primary outcome of the study will be stress level measured by PSS compared among the three groups, at the third time interval that corresponds to the completion of the mindfulness intervention by the experimental group (**Figure 1**). Data will be analyzed using Generalized Linear Mixed Models for repeated measures, considering the baseline levels and within-group comparisons across time. Mixed models have several advantages for such a design: 1) they can be used for modeling non-normal response variables and for modeling discrete responses alike, 2) they account for within subject correlations in repeated measures, 3) utilize all available data even in the presence of missing components, and 4) have greater power by utilizing all available data for more appropriate inferences. The models will include other covariates, such as participant age and sex, and other potential confounders. A demographics questionnaire will collect information about the participants that will also include profession, and level and type of exposure to COVID-19. Post-hoc analyses will

be carried out for specific pairwise comparisons (e.g., Nature+Mindfulness vs Nature only, Nature only vs waitlist control), adjusting for multiple comparisons using the Stepdown Bonferroni method. This method is appropriate for the analysis of the primary outcome (as described), as well as for the secondary outcomes. Selection bias is a limitation in this study, and therefore results will be generalizable to a target population similar to those who participate in this study.

7. **Privacy Interests of the Participants:** The screening will occur only by phone. Only the research team will be screening subjects either in the privacy of their office home or office. Participants will be given a unique number to access REDCap for data entry and will have full control over when and where they engage in this activity.

8. **Data Storage and Confidentiality**
   Self-reported survey electronic data will be collected from participants de-identified. Data will be collected by direct input into the REDCap database management system. Subjects will have a unique identifier that links them to their study groups. They will be given a random unique number to sign on to the database (REDCap) where they input their data directly. Names, phone numbers, and emails will be collected for screening purposes. Data and linking codes will be kept at the NIH and will not be entered into the database. Only the unique subject number will be linked to their groupings and data. The screening data will not be part of the study data and will only be used to determine eligibility. The screening data will be retained for reference under double-lock. The Principal Investigator will oversee the storage and management of data. Identifying information and linkage will be kept in locked cabinets behind locked doors. Database manager (Frank Velez) will have access to the codes but no access to the screening information names, phone numbers, or emails. Subject names, screening information, and unique numbers will not be a part of the data base and will be maintained separately. Only the principal investigator will have access to this information. Co-investigators will have access to REDCap data but not other identifiers. REDCap data will have firewall protection, will be password protected, and only accessed by the study team. Data will be maintained and destroyed based on prevailing NIH guidelines at the appropriate time.

References

1. Lu W, Wang H, Lin Y, Li L. Psychological status of medical workforce during the COVID-19 pandemic: A cross-sectional study. *Psychiatry Res.* 2020;288:112936.
2. Shechter A, Diaz F, Moise N, et al. Psychological distress, coping behaviors, and preferences for support among New York healthcare workers during the COVID-19 pandemic. *Gen Hosp Psychiatry.* 2020;66:1-8.
3. Lai J, Ma S, Wang Y, et al. Factors Associated With Mental Health Outcomes Among Health Care Workers Exposed to Coronavirus Disease 2019. *JAMA Network Open.* 2020;3(3):e203976-e203976.
4. Huang J, Liu F, Teng Z, et al. Care for the psychological status of frontline medical staff fighting against COVID-19. *Clin Infect Dis.* 2020.
5. Santarone K, McKenney M, Elkbuli A. Preserving mental health and resilience in frontline healthcare workers during COVID-19. *Am J Emerg Med.* 2020:S0735-6757(0720)30258-30258.
6. Kisely S, Warren N, McMahon L, Dalais C, Henry I, Siskind D. Occurrence, prevention, and management of the psychological effects of emerging virus outbreaks on healthcare workers: rapid review and meta-analysis. *BMJ.* 2020;369:m1642.
7. Wang S, Xie L, Xu Y, Yu S, Yao B, Xiang D. Sleep disturbances among medical workers during the outbreak of COVID-2019. *Occup Med (Lond).* 2020.
8. Wu K, Wei X. Analysis of Psychological and Sleep Status and Exercise Rehabilitation of Front-Line Clinical Staff in the Fight Against COVID-19 in China. *Med Sci Monit Basic Res.* 2020;26:e924085.
9. Spoormaker VI, Montgomery P. Disturbed sleep in post-traumatic stress disorder: secondary symptom or core feature? *Sleep Med Rev.* 2008;12(3):169-184.
10. Gehrman P, Seelig AD, Jacobson IG, et al. Predeployment Sleep Duration and Insomnia Symptoms as Risk Factors for New-Onset Mental Health Disorders Following Military Deployment. *Sleep.* 2013;36(7):1009-1018.
11. Pigeon WR, Campbell CE, Possemato K, Ouimette P. Longitudinal relationships of insomnia, nightmares, and PTSD severity in recent combat veterans. *J Psychosom Res.* 2013;75(6):546-550.
12. van Liempt S, van Zuiden M, Westenberg H, Super A, Vermetten E. Impact of impaired sleep on the development of PTSD symptoms in combat veterans: a prospective longitudinal cohort study. *Depress Anxiety.* 2013;30(5):469-474.
13. Biddle DJ, Kelly PJ, Hermens DF, Glozier N. The association of insomnia with future mental illness: is it just residual symptoms? *Sleep Health.* 2018;4(4):352-359.
14. Wang HE, Campbell-Sills L, Kessler RC, et al. Pre-deployment insomnia is associated with post-deployment post-traumatic stress disorder and suicidal ideation in US Army soldiers. *Sleep.* 2019;42(2).
15. Hertenstein E, Feige B, Gmeiner T, et al. Insomnia as a predictor of mental disorders: A systematic review and meta-analysis. *Sleep Med Rev.* 2019;43:96-105.

16. West CP, Dyrbye LN, Shanafelt TD. Physician burnout: contributors, consequences and solutions. *J Intern Med.* 2018;283(6):516-529.
17. Lebares CC, Guvva EV, Ascher NL, O'Sullivan PS, Harris HW, Epel ES. Burnout and Stress Among US Surgery Residents: Psychological Distress and Resilience. *J Am Coll Surg.* 2018;226(1):80-90.
18. Hyer L, Boyd S, Scurfield R, Smith D, Burke J. Effects of Outward Bound Experience as an adjunct to inpatient PTSD treatment of war veterans. *J Clin Psychol.* 1996;52(3):263-278.
19. Gelkopf M, Hasson-Ohayon I, Bikman M, Kravetz S. Nature adventure rehabilitation for combat-related posttraumatic chronic stress disorder: a randomized control trial. *Psychiatry Res.* 2013;209(3):485-493.
20. Stigsdotter UK PA, Burls A, Chermaz A, Ferrini, A, Grahn P. Nature-based Therapeutic Interventions. Dordrecht. *Dordrecht: Springer.* 2011.
21. Poulsen DV, Stigsdotter UK, Davidsen AS. "That Guy, Is He Really Sick at All?" An Analysis of How Veterans with PTSD Experience Nature-Based Therapy. *Healthcare (Basel).* 2018;6(2):64.
22. Wagenfeld A, Roy-Fisher, C., Mitchell, C. . Collaborative design: outdoor environments for veterans with PTSD. *Facilities.* 2013;31(9):391-406.
23. Rosenberg RS, Lange W, Zebrack B, Moulton S, Kosslyn SM. An outdoor adventure program for young adults with cancer: positive effects on body image and psychosocial functioning. *J Psychosoc Oncol.* 2014;32(5):622-636.
24. Zebrack B, Kwak M, Sundstrom L. First Descents, an adventure program for young adults with cancer: who benefits? *Support Care Cancer.* 2017;25(12):3665-3673.
25. Mutz M, Müller J. Mental health benefits of outdoor adventures: Results from two pilot studies. *Journal of Adolescence.* 2016;49:105-114.
26. Kabat-Zinn J, University of Massachusetts Medical Center/Worcester. Stress Reduction Clinic. *Full catastrophe living : using the wisdom of your body and mind to face stress, pain, and illness.* Delta trade pbk. reissue. ed. New York, N.Y.: Delta Trade Paperbacks; 2005.
27. Vonderlin R, Biermann M, Bohus M, Lyssenko L. Mindfulness-Based Programs in the Workplace: a Meta-Analysis of Randomized Controlled Trials. *Mindfulness.* 2020.
28. Rusch HL, Rosario M, Levison LM, et al. The effect of mindfulness meditation on sleep quality: a systematic review and meta-analysis of randomized controlled trials. *Ann N Y Acad Sci.* 2019;1445(1):5-16.
29. Goyal M, Singh S, Sibinga EMS, et al. Meditation Programs for Psychological Stress and Well-being: A Systematic Review and Meta-analysis. *JAMA Internal Medicine.* 2014;174(3):357-368.
30. Hofmann SG, Sawyer AT, Witt AA, Oh D. The effect of mindfulness-based therapy on anxiety and depression: A meta-analytic review. *J Consult Clin Psychol.* 2010;78(2):169-183.
31. Eberth J SP. The effects of mindfulness meditation: a meta-analysis. *Mindfulness.* 2012;3(3):174-189.
32. Howells A, Ivtzan I, Eiroa-Orosa FJ. Putting the ‘app’ in Happiness: A Randomised Controlled Trial of a Smartphone-Based Mindfulness Intervention to Enhance Wellbeing. *Journal of Happiness Studies.* 2016;17(1):163-185.

33. Ly KH, Trüschel A, Jarl L, et al. Behavioural activation versus mindfulness-based guided self-help treatment administered through a smartphone application: a randomised controlled trial. *BMJ Open.* 2014;4(1):e003440.
34. van Emmerik AAP, Berings F, Lancee J. Efficacy of a Mindfulness-Based Mobile Application: a Randomized Waiting-List Controlled Trial. *Mindfulness (N Y).* 2018;9(1):187-198.
35. Flett JA HH, Riordan BC, Thompson LM, Conner TS. Mobile mindfulness meditation: a randomised controlled trial of the effect of two popular apps on mental health. *Mindfulness.* 2019;10(5):863-876.
36. Champion L, Economides M, Chandler C. The efficacy of a brief app-based mindfulness intervention on psychosocial outcomes in healthy adults: A pilot randomised controlled trial. *PLoS One.* 2018;13(12):e0209482.
37. Ameli R SN, West C, Luna MJ, Panahi S, Zoosman M, Rusch HL, and Berger A. The effects of a brief mindfulness-based program on stress in healthcare professionals at a biomedical research hospital: a randomized controlled trial. *JAMA Network Open* 2020;In Press.
38. Ameli R SP, Abraham P, Panahi S, Kazman J, Foote F, Deuster P, and Berger A. A Nature-Based Heath Intervention at a Military Healthcare Center: A randomized controlled trial. *In Preparation.* 2020.
39. Capaldi CA, Dopko RL, Zelenski JM. The relationship between nature connectedness and happiness: a meta-analysis. *Front Psychol.* 2014;5:976.
40. Lumber R, Richardson M, Sheffield D. Beyond knowing nature: Contact, emotion, compassion, meaning, and beauty are pathways to nature connection. *PLOS ONE.* 2017;12(5):e0177186.
41. Chiesa A, Serretti A. Mindfulness-Based Stress Reduction for Stress Management in Healthy People: A Review and Meta-Analysis. *The Journal of Alternative and Complementary Medicine.* 2009;15(5):593-600.
42. Shapiro SL AJ, Bishop SR, Cordova M. Mindfulness-Based Stress Reduction for Health Care Professionals: Results From a Randomized Trial. *nternational Journal of Stress Management.* 2005;12(2):164-176.
43. Champion L, Economides M, Chandler C. The efficacy of a brief app-based mindfulness intervention on psychosocial outcomes in healthy adults: A pilot randomised controlled trial. *PLOS ONE.* 2019;13(12):e0209482.
44. Morrison Wylde C, Mahrer NE, Meyer RML, Gold JI. Mindfulness for Novice Pediatric Nurses: Smartphone Application Versus Traditional Intervention. *Journal of Pediatric Nursing.* 2017;36:205-212.
45. Wen L, Sweeney TE, Welton L, Trockel M, Katznelson L. Encouraging Mindfulness in Medical House Staff via Smartphone App: A Pilot Study. *Acad Psychiatry.* 2017;41(5):646-650.
46. Economides M, Martman J, Bell MJ, Sanderson B. Improvements in Stress, Affect, and Irritability Following Brief Use of a Mindfulness-based Smartphone App: A Randomized Controlled Trial. *Mindfulness.* 2018;9(5):1584-1593.

47. Flett JAM, Fletcher BD, Riordan BC, Patterson T, Hayne H, Conner TS. The peril of self-reported adherence in digital interventions: A brief example. *Internet Interventions.* 2019;18:100267.
48. Laurie J, Blandford A. Making time for mindfulness. *International Journal of Medical Informatics.* 2016;96:38-50.
49. Cohen S, Kamarck T, Mermelstein R. A Global Measure of Perceived Stress. *Journal of Health and Social Behavior.* 1983;24(4):385-396.
50. Nielsen MG, Ørnbøl E, Vestergaard M, et al. The construct validity of the Perceived Stress Scale. *J Psychosom Res.* 2016;84:22-30.
62. Ameli R, Sinaii N, Luna MJ, Cheringal J, Gril B, Berger A. The National Institutes of Health measure of Healing Experience of All Life Stressors (NIH-HEALS): Factor analysis and validation. *PLOS ONE.* 2018;13(12):e0207820.
63. Sloan DH, BrintzenhofeSzoc K, Kichline T, et al. An assessment of meaning in life-threatening illness: development of the Healing Experience in All Life Stressors (HEALS). *Patient Relat Outcome Meas.* 2017;8:15-21.
